# Comparison of Five Oxidative Stress Biomarkers in Vegans and Omnivores from Germany and Finland

**DOI:** 10.3390/nu14142918

**Published:** 2022-07-16

**Authors:** Stefan Dietrich, Anna-Liisa Elorinne, Nick Bergau, Klaus Abraham, Tilman Grune, Juha Laakso, Daniela Weber, Cornelia Weikert, Bernhard H. Monien

**Affiliations:** 1 Department of Food Safety, German Federal Institute for Risk Assessment, 10589 Berlin, Germany; nick.bergau@bfr.bund.de (N.B.); klaus.abraham@bfr.bund.de (K.A.); cornelia.weikert@bfr.bund.de (C.W.); bernhard.monien@bfr.bund.de (B.H.M.); 2 School of Applied Educational Science and Teacher Education, University of Eastern Finland, 80101 Joensuu, Finland; anna-liisa.elorinne@uef.fi; 3 Department of Molecular Toxicology, German Institute of Human Nutrition, Potsdam-Rehbruecke (DIfE), 14558 Nuthetal, Germany; scientific.director@dife.de (T.G.); daniela.weber@dife.de (D.W.); 4 Clinical Research Institute HUCH, 00280 Helsinki, Finland; juha.laakso@alumni.helsinki.fi

**Keywords:** oxidative stress, malondialdehyde, protein carbonyls, 3-nitrotyrosine, 8-hydroxy-2′-deoxyguanosine, 8-iso-prostaglandin F2α, vegan diet, omnivorous diet

## Abstract

When the amount of reactive oxygen species produced by human metabolism cannot be balanced by antioxidants, this phenomenon is commonly referred to as oxidative stress. It is hypothesised that diets with high amounts of plant food products may have a beneficial impact on oxidative stress status. However, few studies have examined whether a vegan diet is associated with lower oxidative stress compared to an omnivorous diet. The present cross-sectional study aimed to compare the levels of five oxidative stress biomarkers in vegans and omnivores. Data of 36 vegans and 36 omnivores from Germany and of 21 vegans and 18 omnivores from Finland were analysed. HPLC coupled with mass spectrometry or fluorescence detection and ELISA methods were used to measure the oxidative stress biomarkers malondialdehyde (MDA), protein carbonyls and 3-nitrotyrosine in plasma and 8-hydroxy-2′-deoxyguanosine (8-OHdG) and 8-iso-prostaglandin F2α (8-iso-PGF2α) in 24 h urine. Analyses of variance and covariance, considering potential confounders, were used. Vegans and omnivores showed no differences in MDA and protein carbonyl concentrations. In Finnish but not in German vegans, the concentrations of 3-nitrotyrosine were lower compared to those in omnivores (*p* = 0.047). In Germany, vegans showed lower excretion levels of 8-iso-PGF2α than omnivores (*p* = 0.002) and with a trend also of 8-OHdG (*p* = 0.05). The sensitivity analysis suggests lower 8-iso-PGF2α excretion levels in women compared to men, independently of the dietary group. The present study contributes to expanding our knowledge of the relationship between diet and oxidative stress and showed that 3-nitrotyrosine, 8-OHdG and 8-iso-PGF2α tended to be lower in vegans. Furthermore, studies are recommended to validate the present findings.

## 1. Introduction

An imbalance between oxidants and antioxidants with the formation of reactive oxygen species (ROS) in cells and tissues is characterised as a metabolism in the state of oxidative stress [1]. ROS arise through many metabolic processes of multifactorial origin [2]. The main endogenous sources of ROS are mitochondrial respiration processes and redox enzymes [2,3]. The reaction of ROS with cellular components such as proteins, lipids and DNA promotes the formation of biomarkers for oxidative stress [4]. Since the majority of ROS are extremely reactive and thus have a very short half-life, the concentration of biomarkers of oxidative stress in blood and urine is often used to evaluate an individual’s oxidative stress status [2]. Reliable biomarkers of oxidative stress are: (1) malondialdehyde (MDA), an indicator of lipid peroxidation [5]; (2) protein carbonyls, an indicator of conversion reactions of amino acid side-chains (e.g., deamination or imine formation) [6]; (3) 3-nitrotyrosine, an indicator of tyrosine nitration often associated with inflammation [5], (4) 8-hydroxy-2′-deoxyguanosine (8-OHdG), an indicator of DNA nucleobase modifications by hydroxyl radicals that showed associations with cancer and cardiovascular disease [7,8]; and (5) 8-iso-prostaglandin F2α (8-iso-PGF2α), an indicator of free radical-catalysed peroxidation of arachidonic acid [5].

Studies indicate that elevated levels of these biomarkers are tightly linked with an unbalanced diet, unhealthy lifestyle and chronic inflammatory diseases [5,9]. The formation of oxidative stress biomarkers may be prevented by antioxidants that act as free radical scavengers or as inducers of antioxidative endogenous systems and thus as antagonists of ROS [2]. Antioxidants from plants reacting directly with ROS are carotenoids (xanthophylls and carotenes) and vitamins (vitamin E and C), whereas polyphenols (e.g., phenolic acids, flavonoids, anthocyanins and lignans) may also function indirectly by the induction of the nuclear factor erythroid 2-related factor 2 (Nrf2)-antioxidant response element (ARE) pathway [10,11]. In this context, it has been hypothesised that diets with high intakes of different antioxidants may have beneficial effects, which may be reflected by lower levels of oxidative stress biomarkers [9,12,13]. A diet rich in antioxidants is therefore considered as a preventive strategy to counteract high ROS levels. Accordingly, several observational and intervention studies observed that the Mediterranean diet, the Dietary Approaches to Stop Hypertension (DASH) diet or dietary patterns with a high proportion of fruits and vegetables are associated with lower levels of several oxidative stress biomarkers, including MDA, F2-isoprostane, and 8-OHdG [9]. Contrary to this, Western and fast food diets tended to increase concentrations of oxidative stress biomarkers [9]. In some studies that compared vegetarian with omnivorous diets, vegetarians showed lower MDA and protein carbonyl concentrations than comparison groups [13].

Despite these interesting findings, the above-mentioned biomarkers of oxidative stress have not been adequately studied in terms of diet and for a vegan diet in particular. Therefore, the present study aimed to compare a comprehensive spectrum of five biomarkers of oxidative stress (MDA, protein carbonyls, 3-nitrotyrosine, 8-OHdG, and 8-iso-PGF2α) between dietary groups of vegans and omnivores from Germany and Finland.

## 2. Materials and Methods

### 2.1. Study Populations

The present study analysed data on biomarkers of oxidative stress in participants from the following two independently designed cross-sectional studies: (I) 36 vegans and 36 omnivores of the Risks and Benefits of a Vegan Diet (RBVD) study from Germany [14] and (II) 22 vegans and 19 omnivores of a study from Finland [15]. One vegan and one omnivore had to be omitted from the Finnish study due to missing blood samples. The primary research question in the RBVD study was on bone health in vegans compared to omnivores [16] and secondary research questions were among others on nutrient status and dietary intake [14]. The goal of the Finnish study was to compare the dietary intake and nutrient status of vegans and omnivores [15]. The RBVD study was conducted in the period from January to July 2017 and the Finnish study in November 2011. In the RBVD study, vegans and omnivores were sex- and age-matched, while in the Finnish study matching was performed according to age only. In both studies, vegan participants followed their diet for at least one year [14,15]. The RBVD study included participants with an age between 30 and 60 years, and exclusion criteria were a body mass index (BMI) ≥ 30, cardiovascular disease, type two diabetes mellitus, cancer, pregnancy, breastfeeding, and current infection [14]. The Finnish study included participants with an age between 18 and 50 years, healthy condition, and nonusers of regular medications (except oral contraceptives or hormone replacement therapy) [15].

The RBVD study was approved by the Ethics Committee of the Charité-Univer-sitätsmedizin Berlin (no. EA4/121/16) [14]. The Finnish study received the approval of the Ethical Committee of Kuopio University Hospital (69//2011) [15]. The studies were conducted according to the Declaration of Helsinki. All participants provided their written consent to participate in these studies [14,15] and in the usage of the biomatrices for the analysis of laboratory parameters.

### 2.2. Blood and Urine Collection and Laboratory Analysis

The two studies collected fasting peripheral venous blood samples during visits at the respective study centres. Blood samples were fractionated into plasma or serum and stored in freezers at −80 °C in the RBVD study and at −70 °C in the Finnish study until analysis. The participants of the RBVD study collected urine over 24 h in provided preservative-free plastic containers on the day before visiting the study center. Collected 24 h urine samples were aliquoted and stored at −80 °C until analysis. In the Finnish study, 24 h urine samples were not available for measurement of the urinary biomarkers 8-OHdG and 8-iso-PGF2α.

MDA, protein carbonyls, and 3-nitrotyrosine concentrations were measured in all plasma samples at the German Institute of Human Nutrition Potsdam-Rehbruecke (DIfE) in 2020. Samples of vegans and omnivores of both studies were randomly analysed at the same time. MDA was detected by fluorescence after derivatisation with thiobarbituric acid and separation using reversed-phase high performance liquid chromatography (RP-HPLC) as described by Wong et al. [17] with some modifications [18]. Non-commercial, in-house enzyme-linked immunosorbent assays (ELISAs) were used to analyse protein carbonyls [16] and 3-nitrotyrosine [19], respectively. In the RBVD study, 8-iso-PGF2α and 8-OHdG excretion levels were determined in 24 h urine samples at the German Federal Institute for Risk Assessment (BfR). The analytes were quantified using isotope-dilution liquid chromatography tandem-mass spectrometry (LC-MS/MS) analyses. The 8-iso-PGF2α analysis was based on the solid-phase extraction method of Dahl and Breeman [20], whereas the analysis of 8-OHdG followed a dilute-and-shoot approach. In addition, cyanoethyl mercapturic acid (CYMA), a short-term biomarker of smoking, was determined in 24 h urine samples at the BfR using an LC-MS/MS method. A detailed description of the laboratory analyses can be found in the Supplementary Material File S1.

### 2.3. Assessment of Diet and Lifestyle Characteristics

In the two studies, dietary habits were recorded using three-day weighed food records [14,15]. In the RBVD study, participants weighed their foods [14], whereas participants in the Finnish study used household measures [15]. Based on the data of the three-day weighed food records, the intake of macronutrients and alcohol was calculated using the German Nutrient Database (BLS, Bundeslebensmittelschlüssel) Version 3.02 in the RBVD study [14] and the Finnish Nutrica^®^ software version 2.5 in the Finnish study [15].

In the RBVD study, trained personnel conducted anthropometric measurements of weight and height on participants wearing only light underwear. Computer-based questionnaires were used to assess data including age, sex, physical activity (PA), smoking status and further lifestyle and health characteristics. In the Finnish study, a computer-based questionnaire was used to assess data including participants’ age, sex, weight, height, smoking status, alcohol consumption, PA and several other health and lifestyle characteristics.

In the two studies, PA was dichotomised based on a standard metabolic equivalent of task (MET) to represent inactive (≤33.75 MET h per week) and active (>33.75 MET h per week) participants according to the “recreational index” of the InterAct Consortium [21]. In the RBVD study, MET h per week was calculated as the sum duration of walking, cycling and sports (averaged for summer and winter, in h/week) and multiplied with standard metabolic equivalent of task (MET) estimates (3.0 for walking and 6.0 for cycling and sports). In the Finnish study, MET h per week was calculated as the sum duration of light and heavy PA (h/week) which was multiplied with standard metabolic equivalent of task (MET) estimates (3.0 for light PA and 6.0 for heavy PA).

### 2.4. Statistical Analysis

Participants’ characteristics were reported using mean and standard deviation (SD) for normally distributed variables, median and interquartile range (IQR) for non-normally distributed variables, and relative percentages for categorical variables.

To investigate potential differences between dietary groups in mean concentrations or mean daily excretion levels of oxidative stress biomarkers, an analysis of variance (ANOVA) for an unadjusted model (Model 1) was calculated. Additionally, multivariable adjusted analyses of covariance (ANCOVA) were calculated using two sets of potential confounders (Model 2 and 3). Model 2 was adjusted for age (years), sex (women, men), and BMI (kg/m^2^). Model 3 was adjusted for age, sex, BMI and additionally for smoking status (yes or no), PA (inactive or active), alcohol consumption (g/day) and total energy intake (kcal/d). In the RBVD study, an additional model (Model 4) was calculated including confounders of Model 3 but smoking status was replaced by excretion of CYMA. The oxidative stress biomarkers were skewed and to approximate normal distribution, the bio-markers were logarithmically transformed for the ANOVA and ANCOVA analyses. Afterwards, the estimators of logarithmically transformed biomarkers were back-transformed and expressed as geometric means and 95%-confidence intervals (95%-CI). Protein carbonyls in the Finnish study showed normal distribution and thus a linear regression analysis was applied to protein carbonyls. In the Finnish study, four omnivores had missing data for energy intake and macronutrients intake, three of them also for PA, and two of them also for BMI and smoking status. These omnivores were excluded from the ANCOVA analysis of the respective models.

Boxplots were computed to visualise concentrations of the oxidative stress biomarkers stratified for diet, sex and smoking. Scatterplots were computed to visualise potential associations between oxidative stress biomarkers and CYMA. To investigate correlations between biomarkers of oxidative stress, Spearman correlations were calculated. Test findings with *p*-values of <0.05 were considered statistically significant. All statistical analyses were performed using SAS software, version 9.4 (SAS Institute, Cary, NC, USA) and R software (version 4.0.3, R Foundation for Statistical Computing, Vienna, Austria).

## 3. Results

The baseline characteristics of the two study populations differed only slightly (Table 1). In the RBVD study, the sex distribution was equal, while the proportion of women was higher in the Finnish study (vegans: 71.4%, omnivores 55.6%). The participants in the RBVD study were slightly older (vegans: 37.5 years, omnivores 38.5 years) than those in the Finnish study (vegans: 33.0 years, omnivores 34.0 years). The proportion of smokers was low in both studies. The participants of the RBVD study were more physically active than the ones of the Finnish study. The energy intake was higher among participants of the RBVD study (vegans: 2297 kcal/day, omnivores: 2386 kcal/day) than that of the Finnish study with lowest values among Finnish omnivores (vegans: 2135 kcal/d, omnivores: 1992 kcal/d). No dietary data were available for four Finnish omnivores. In the two studies, the protein intake in vegans (RBVD: 72.2 g/day; Finnish: 70.7 g/day) was lower than in omnivores (RBVD: 86.3 g/day, Finnish: 93.5 g/day), whereas the fibre intake in vegans (RBVD: 45.6 g/day, Finnish: 39.9 g/day) was higher than in omnivores (RBVD: 23.7 g/day, Finnish: 25.1 g/day). The carbohydrate intake was lowest in omnivores from Finland with a mean intake of 180.6 g/day, respectively. The fat intake was highest in omnivores of the RBVD study with 104.1 g/day.

### 3.1. Oxidative Stress Biomarkers

**Figure 1:**
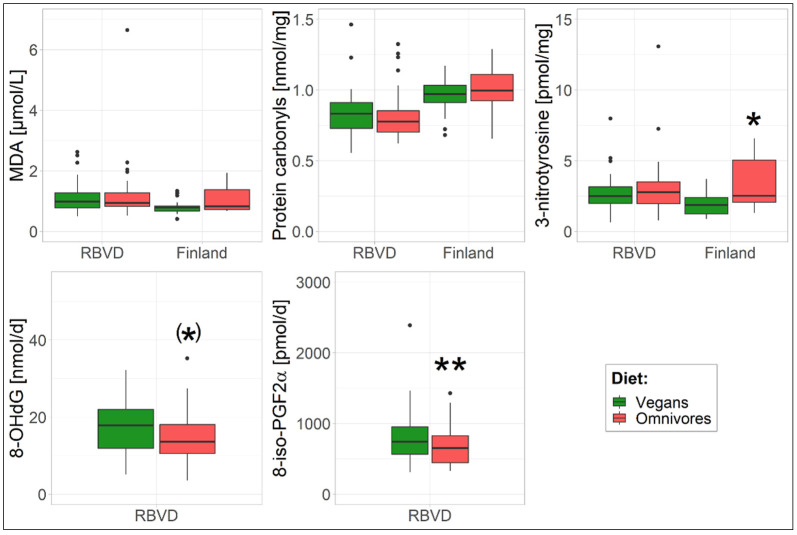
Boxplots of oxidative biomarkers in the three study populations. Dots represent extreme values. ^(^*^)^ *p* with trend, * *p*  <  0.05; ** *p*  <  0.01; *p*-values of model 3 in Table 2 are reported.

Overall, no significant differences for concentrations of MDA and protein carbonyls were observed between vegans and omnivores in the two study populations (Table 2, Figure 1). However, in the Finnish study, the concentrations of protein carbonyls in vegans and omnivores were approximately 15% higher than the ones in the RBVD study, but without significant differences between the two dietary groups. In the RBVD study, the concentrations of 3-nitrotyrosine were not significantly different between vegans and omnivores (Table 2, Figure 1). In contrast, a significantly lower 3-nitrotyrosine concentration (*p* = 0.04, Table 2) was observed in Finnish vegans (1.8 pmol/mg, 95%-CI 1.47–2.21) compared to Finnish omnivores (3.14 pmol/mg, 95%-CI 2.41–4.09) considering potential confounding factors. The 3-nitrotyrosine concentrations of the Finnish vegans tended to be lower than the ones of the vegans from the RBVD study (2.44 pmol/mg, 95%-CI 2.08–2.85).

The daily urinary excretion of 8-OHdG and 8-iso-PGF2α in 24 h urine was measured in the RBVD study only (Table 2, Figure 1). The daily 8-iso-PGF2α excretion of vegans was significantly lower (*p* = 0.002, 662 pmol/d, 95%-CI 585–749 pmol/d) compared to that of omnivores (742 pmol/d, 95%-CI 656–839 pmol/d) in the multivariable adjusted model. In addition, vegans also showed a trend of lower 8-OHdG excretion (*p* = 0.05, Table 2) than omnivores in the multivariable adjusted model.

A significant correlation between oxidative stress biomarkers (Figure S1) was found in the RBVD study between excretion levels of 8-iso-PGF2α and 8-OHdG (RBVD: vegans r = 0.40, *p* = 0.02, omnivores r = 0.38, *p* = 0.02) only. The other biomarkers were not correlated with each other, neither in the RBVD nor in the Finnish study (Figures S1 and S2).

### 3.2. Sensitivity Analysis

**Figure 2:**
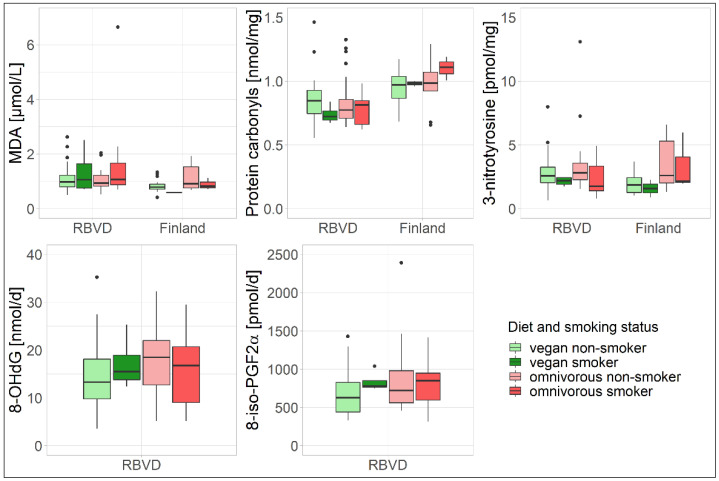
Boxplots of the oxidative damage biomarkers stratified by dietary group and smoking status in the RBVD and the Finnish study. Dots represent extreme values. No significant differences were observed for the interaction term of diet with smoking (Tables S1 and S2).

The sensitivity analysis indicated nearly no effect of smoking on oxidative stress biomarkers in either the RBVD study or in the Finnish study (Tables S1 and S2, Figure 2). In the RBVD study, and only MDA showed a borderline significant association with smoking status (*p* = 0.05, Table S1) and a significant association with CYMA (*p* < 0.01, Table S3), regardless of the dietary group. The regression model with CYMA (R^2^ = 0.15, Table S3) instead of smoking status (R^2^ = 0.02, Table S1) showed elevated explained variation in MDA. When the confounder smoking status was replaced by CYMA in the ANCOVA analysis, the MDA concentrations among vegans were significantly higher than in omnivores (*p* = 0.04, Table S4). However, when the one omnivore with an extreme MDA concentration of higher than 6 µmol/L was excluded, no significant difference was observed anymore. Moreover, the scatter plot of MDA and CYMA (Figure S3) suggests only minor difference in MDA concentration due to CYMA. An interaction effect of diet and smoking on the other biomarkers of oxidative stress was not observed in either the RBVD study or in the Finnish study (Tables S1–S3, Figure 2 and Figure S3). Exclusion of smokers did not change the overall findings for oxidative stress biomarkers regarding the differences between vegans and omnivores (Table S5).

**Figure 3:**
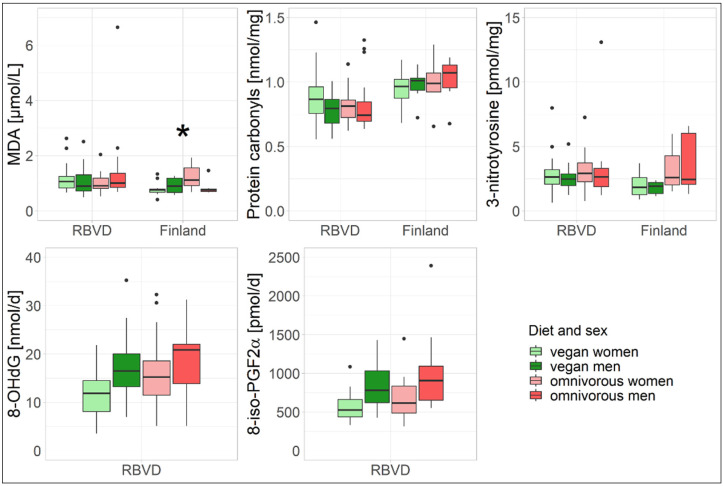
Boxplots of the oxidative damage biomarkers stratified by dietary group and sex in the RBVD and the Finnish study. Dots represent extreme values. * *p*  <  0.05; *p*-values of the interaction term of diet with sex from Tables S6 and S7 are reported.

The sensitivity analysis suggested an effect of sex on some oxidative stress biomarkers (Figure 3, Tables S6 and S7). In the Finnish study, women had significantly higher MDA concentrations than men (*p* = 0.04, Table S7); however, it seems to be mainly driven by women with an omnivorous diet (Figure 3). In the RBVD study, men showed significantly higher 8-iso-PGF2α excretion levels (Figure 3, Table S6, *p* = 0.01, vegan: 770 pmol/d, 95%-CI 640–926; omnivore: 907 pmol/d, 95%-CI 755–1091) than women (vegan: 540 pmol/d, 95%-CI 457–638; omnivore: 640 pmol/d, 95%-CI 542–756), regardless of the dietary group. There were also indications of lower excretion levels of 8-OHdG among women compared to men (Figure 3 and Table S8), but the difference was not statistically significant (*p* = 0.22, Tables S6 and S8). In addition, the sensitivity analysis did not indicate differences in concentrations and excretion levels of oxidative stress biomarkers due to PA (data not shown).

## 4. Discussion

Plant-based diets are becoming increasingly popular in Western societies. The present study pursued the question of whether a vegan diet is associated with lower oxidative stress using five different biomarkers of oxidative stress. In Finnish vegans, lower concentrations of 3-nitrotyrosine were observed, whereas excretion levels of 8-OHdG and 8-iso-PGF2α were lower in German vegans compared to German omnivores. The two dietary groups showed similar concentrations of MDA and protein carbonyls.

Elevated levels of oxidative stress biomarkers are indicative of a metabolism in the state of oxidative stress. The formation of biomarkers of oxidative stress is triggered by ROS. ROS are detoxified by the so-called antioxidant defence system that includes diverse antioxidants serving as radical scavengers [2]. Antioxidants of dietary intake are of particular interest for research, as they may preventively contribute to reducing oxidative stress [2]. The most important antioxidants in plants can be divided into six groups, i.e., (1) vitamins, (2) catechins, (3) isothiocyanates, (4) capsaicinoids and casinoids, (5) carotenoids, and 6) polyphenols (e.g., stilbenes, flavonoids and phenolic acids). They work as antioxidants by either directly scavenging ROS or preventing the activation of nuclear factor κ-light-chain-enhancer of activated B cells (NF-κB) and thereby reduce the cellular inflammatory response [22]. Because previous studies yielded controversial results when evaluating the antioxidant capacity of a limited number of single foods or nutrients, a more recent approach was developed to examine whole diets to consider the systemic complex processes involved in oxidative stress [9,12,13]. Hence, the present study compared biomarker levels in participants following a vegan or an omnivorous diet and observed that the levels of three out of five oxidative stress biomarker were lower in vegans than in omnivores.

MDA is one of the most frequently examined biomarkers of oxidative stress formed after the oxidation of polyunsaturated fatty acids [2]. Several studies observed lower MDA concentrations in participants following a vegetarian or Mediterranean diet compared to other diets [9,13]. However, other studies observed no effect of a vegetarian or Mediterranean diet on MDA concentrations [9,13]. A previous study that included a vegan dietary group observed even higher MDA concentrations in vegans than in the comparison groups [23]. In the present study, the two study populations showed no differences in MDA concentrations between vegans and omnivores. Considering CYMA as a confounding factor in the RBVD study instead of smoking status, vegans of the RBVD study showed slightly higher MDA concentrations than omnivores. However, this finding should be interpreted with caution as few participants were smokers and the contribution of CYMA to the overall variability of MDA was small. In addition, a recent meta-analysis showed that smoking status had no effect on urinary MDA concentrations [24].

Likewise, the present study observed no differences between protein carbonyl levels in vegans and omnivores. So far, two studies have examined protein carbonyls in relation to diet. One of the studies observed no differences between vegetarians and omnivores [25]. The other study observed lower levels in vegetarians of older ages (60–70 years) compared to omnivores of older ages (60–70 years) [26]. Surprisingly, protein carbonyl concentrations in participants from Finland were higher than those from Germany. Since the two study populations included healthy participants with similar characteristics, one can only speculate about the cause, e.g., genetic differences at the population level, differences in the environment, lifestyle or in the composition and quality of the diet.

The present study observed inconsistent findings for 3-nitrotyrosine with significantly lower values in Finnish vegans compared to omnivores, whereas this finding was not confirmed in the RBVD study. At elevated concentrations of nitrogen species, 3-nitrotyrosine is formed through the post-translational modification of tyrosine residues by nitric oxide derived oxidants [27,28]. Several studies found associations of 3-nitrotyrosine with inflammatory and neurodegenerative diseases [27,28], but there are almost no studies examining 3-nitrotyrosine in relation to diet. In two previous studies, vegans and vegetarians showed higher blood levels of nitrite and nitrate than omnivores [23,29]. Some plant-based foods (e.g., green leafy vegetables) are a rich source of inorganic nitrate [30], but a high dietary intake of nitrate does not seem to be associated with an increase in circulating 3-nitrotyrosine levels [31]. In an animal study, a high-fat diet was associated with higher levels of protein-bound 3-nitrotyrosine in New Zealand obese mice [32]. Obesity is accompanied by chronic inflammation and oxidative stress [33]. However, the present study analysed data of non-obese participants only and observed no correlation between the dietary intake of total fat and 3-nitrotyrosine concentration in either vegans or in omnivores (data not shown). Because of the lack of current evidence, it is imperative to conduct further research to validate the results of 3-nitrotyrosine presented here.

The present study observed indications of lower excretion levels of the two urinary biomarkers, namely 8-OHdG and 8-iso-PGF2α in vegans of the RBVD study. In some previous studies, lower 8-OHdG concentrations were observed in participants with high adherence to a Mediterranean diet [9]. To the best of our knowledge, there has been no research of 8-OHdG in studies on vegan or vegetarian diets so far. However, some studies examined the link between other biomarkers of oxidative genome damage and a vegetarian or vegan diet, but with inconclusive results [13]. The biomarker 8-iso-PGF2α is an isoprostane produced by the radical-catalysed oxidation of arachidonic acid [5]. To date, there does not seem to be any studies that have examined the 8-iso-PGF2α status in vegans or vegetarians. A systematic review indicated that high adherence to the Mediterranean diet is associated with lower or no differences in F2-isoprostane levels measured in plasma or urine, depending on the study [9]. Interestingly, in the present study, women had significantly lower 8-iso-PGF2α levels than men. Lower excretion levels of 8-iso-PGF2α in women compared to men were also observed in a previous study [34], but other studies observed no differences [35] or suggested the opposite [36,37]. However, these studies are only comparable to a limited extent with the present study, since their biomatrices and analytical methods differed.

Investigating the causes of the observed differences in oxidative stress biomarkers represents a significant challenge due to the underlying complex metabolic pathways involved. Due to the cross-sectional design and the limited statistical power of the present study, one can only speculate about possible causes. Antioxidants in the diet (e.g., vitamin A, C and E and carotenoids) are part of the antioxidant defence system and may thus contribute to lower concentrations of ROS [2]. A high proportion of dietary antioxidants may be attributed to vegetarian or vegan diets. However, data on antioxidant levels in plant-based diets is contradictory, with higher, lower or equivalent levels of antioxidants in a vegetarian diet compared to an omnivorous diet [13,38]. The two study populations investigated here even showed partly poorer antioxidant status (e.g., carotenoids and vitamin E) in the blood of vegans than of omnivores [14,15]. When looking at the diet, the estimated dietary intake of antioxidants (e.g., carotenoids, vitamin C and E) from the 3-day weighed food records was higher in vegans compared to omnivores in the RBVD study, whereas in the Finnish study it was partly lower in vegans compared to omnivores [14,15]. However, some participants in both studies also took supplements that included antioxidants [14,15]. Some dietary antioxidants, mainly endogenous antioxidants such as glutathione, are involved in the reduction of ROS species and can thus influence concentrations of oxidative stress biomarkers. An effect on oxidative stress is also assumed for lifestyle factors such as smoking, PA and alcohol consumption [39]. In the present study, these confounders hardly differed between vegans and omnivores. Furthermore, inflammatory processes are also related to oxidative stress and may also influence the concentrations of oxidative stress biomarkers [13]. However, no significant differences in inflammatory biomarkers were observed in the RBVD study between vegans and omnivores [16]. In summary, only a few of the many factors that may contribute to the observed levels of oxidative stress biomarkers were listed here. The extent to which the diet and individual dietary components are causally related to the biomarker levels cannot be investigated with the cross-sectional RBVD and Finnish study. Hence, the observed findings should be taken with caution.

A strength of the present study is the simultaneous investigation of biomarkers of oxidative stress in samples from Finland and Germany using the same established laboratory and statistical analyses. This allowed for a cross-population, comparative analysis of the potential differences of these biomarkers in vegans and in omnivores for whom such investigations are relatively rare to date. Accordingly, three oxidative stress biomarkers were determined in plasma samples in two independent study populations. In addition, 24 h urine samples from the RBVD study were used to monitor the total daily excretion of two other biomarkers of oxidative stress. Unfortunately, no 24 h urine samples were available for the Finnish study. In addition, potential confounders, which were assessed under high qualitative standards, could also be considered. Moreover, sensitivity analyses were performed to investigate the influence of potential confounders. The use of three-day weighed food records to estimate the energy intake presented the advantage that vegan-specific foods were also recorded, which may not typically be captured with a food frequency questionnaire.

The present findings are limited by the small study populations. The small study size and potential multicollinearity effects due to different metabolic pathways involved in oxidative stress also make it inadvisable to investigate the association of the five biomarkers with other biomarkers (antioxidants and inflammatory markers). The two studies were independently designed resulting in different assessments of PA. This disadvantage could be compensated for by using the recreational index. Moreover, the cross-sectional design of the two studies does not allow for causal inferences. In addition, only middle-aged people with a healthy general condition were included; therefore, the results may not be generalisable to other populations (e.g., unhealthy, other age, different ethnicity).

## 5. Conclusions

The present study contributes to expanding our knowledge of the relationship between a vegan diet and oxidative stress by comparing five biomarkers in vegans and omnivores in two independent study populations. The vegan diet did not appear to be associated with lower MDA and protein carbonyls levels, the levels of 3-nitrotyrosine, 8-OHdG and 8-iso-PGF2α tended to be lower in vegans in the respective study populations. It would certainly be interesting to identify which food components influence the concentrations of the biomarkers in order to be able to provide nutritional advice. However, the concentrations of oxidative stress biomarkers are influenced by multiple factors that hamper such investigations (endogenous antioxidants, metabolic turnover rates, multicollinearity effects, etc.). Due to the present study design, limited statistical power and the unavailability of some data on endogenous antioxidants, it did not seem appropriate to carry out those statistical analyses. First, the present findings need to be validated by further studies. If confirmed, the underlying metabolic pathways should be identified to understand how diet is associated with the respective biomarkers of oxidative stress so that reliable targeted dietary recommendations can be made in the future.

## Figures and Tables

**Table 1:** Characteristics of the RBDV and the Finnish study stratified by dietary group.

Characteristics	RBVD Study		Finnish Study	
	Vegans (*n* = 36)	Omnivores (*n* = 36)	Vegans (*n* = 21)	Omnivores (*n* = 18)
Duration of diet (years)	4.8 (3.1–8.7)	-	8.0 (6.0–11.0)	-
Women, n (%)	18 (50.0)	18 (50.0)	15 (71.4)	10 (55.6)
Age (years)	37.5 (32.5–44.0)	38.5 (32.0–46.0)	33.0 (28.0–38.0)	34.0 (28.0–39.0)
BMI (kg/m^2^) ^a^	22.9 ± 3.2	24.0 ± 2.1	22.0 ± 2.0	22.7 ± 2.5
Physical activity, n (%) ^a^				
Inactive	5 (13.9)	4 (11.1)	19 (90.5)	14 (77.8)
Active	31 (86.1)	32 (88.9)	2 (9.5)	1 (5.6)
Smoking status, n (%) ^a^				
Non-smoker	32 (88.9)	27 (75.0)	19 (90.5)	13 (72.2)
Smoker	4 (11.1)	9 (25.0)	2 (9.5)	3 (16.7)
Alcohol consumption (g/day)	0.1 (0–3.3)	1.3 (0–11.2)	0 (0–4.1)	1.9 (0–5.9)
Energy intake (kcal/day) ^a^	2297 (1800–2870)	2386 (2081–2737)	2135 (1888–2600)	1992 (1730–2383)
Protein (g/day) ^a^	72.2 (54.9–91.8)	86.3 (71.4–107.0)	70.7 (54.5–90.4)	93.5 (76.7–110.8)
Fat (g/day) ^a^	85.7 (63.6–111.1)	104.1 (87.8–143.3)	88.1 (64.2–114.5)	90.3 (74.2–128.6)
Carbohydrates (g/day) ^a^	258.7 (211.5–371.2)	230.3 (199.3–291.0)	257.7 (203.4–306.6)	180.6 (131.6–238.0)
Fibre (g/day) ^a^	45.6 (33.7–56.4)	23.7 (18.6–29.9)	39.9 (32.6–50.5)	25.1 (22.7–42.0)

Data are reported as percentage, mean ± SD for normally distributed or median (IQR) for non-normally distributed variables. ^a^ In the Finnish study, four omnivores had missing data for energy intake and macronutrients intake, three of them also for PA, and two of them for BMI and smoking status. Abbreviations: BMI, body mass index.

**Table 2:** Biomarkers of oxidative damage according to vegan or omnivorous diet in the RBVD and the Finnish study.

Biomarker	Model	RBVD Study			Finnish Study		
		Vegans (*n* = 36)	Omnivores (*n* = 36)	*p*-Value	Vegans (*n* = 21)	Omnivores (*n* = 18)	*p*-Value
MDA [µmol/L]	1	1.04 (0.90–1.20)	1.08 (0.94–1.25)	0.73	0.79 (0.69–0.91)	0.97 (0.83–1.13)	0.054
	2	1.04 (0.90–1.20)	1.08 (0.94–1.26)	0.99	0.80 (0.69–0.92)	1.00 (0.85–1.17)	0.06
	3 ^a^	1.07 (0.93–1.24)	1.05 (0.91–1.21)	0.30	0.79 (0.69–0.90)	0.97 (0.81–1.15)	0.18
Protein carbonyl ^b^ [nmol/mg]	1	0.82 (0.77–0.87)	0.81 (0.75–0.86)	0.76	0.95 (0.89–1.02)	1.01 (0.94–1.08)	0.26
	2	0.82 (0.76–0.88)	0.81 (0.75–0.86)	0.73	0.96 (0.89–1.02)	1.01 (0.93–1.08)	0.61
	3 ^a^	0.81 (0.76–0.87)	0.81 (0.76–0.87)	0.15	0.96 (0.90–1.02)	0.95 (0.87–1.03)	0.34
3-nitrotyrosine [pmol/mg]	1	2.53 (2.16–2.96)	2.72 (2.32–3.18)	0.51	1.81 (1.48–2.22)	2.98 (2.39–3.72)	**0.002**
	2	2.48 (2.12–2.91)	2.77 (2.36–3.25)	0.57	1.80 (1.45–2.25)	3.06 (2.38–3.94)	**0.04**
	3 ^a^	2.44 (2.08–2.85)	2.82 (2.41–3.30)	0.23	1.80 (1.47–2.21)	3.14 (2.41–4.09)	**0.04**
8-OHdG [nmol/d]	1	13.4 (11.5–15.7)	16.0 (13.7–18.7)	0.12	**-**	**-**	**-**
	2	13.5 (11.6–15.7)	15.8 (13.6–18.5)	**0.04**	**-**	**-**	**-**
	3	13.4 (11.5–15.6)	16.0 (13.7–18.6)	0.05	**-**	**-**	**-**
8-iso-PGF2α [pmol/d]	1	645 (564–737)	762 (666–871)	0.08	**-**	**-**	**-**
	2	657 (582–741)	748 (663–845)	**<0.001**	**-**	**-**	**-**
	3	662 (585–749)	742 (656–839)	**0.002**	**-**	**-**	**-**

Oxidative stress biomarkers are reported as geometric mean (95%-CI). ^a^ In the Finnish study, five omnivores had missing values of confounders and were excluded in Model 3. ^b^ In the Finnish study, protein carbonyl values represent arithmetic means. Model 1: unadjusted; Model 2: adjusted for age, sex, BMI; Model 3: adjusted for age, sex, BMI, smoking status, physical activity, alcohol consumption, total energy intake. Values of *p* < 0.05 were highlighted with bold numbers. Abbreviation: MDA, malondialdehyde; 8-OHdG, 8-hydroxy-2′-deoxyguanosine; 8-iso-PGF2α, 8-iso-prostaglandin F2α.

## Data Availability

Data generated and analysed during the RBVD and Finnish study are not publicly available due to provisions of the data protection regulations and written agreements with participants that the data are not given to the third parties.

## Supplementary Materials

The following supporting information can be downloaded at: https://www.mdpi.com/article/10.3390/nu14142918/s1, File S1: Detailed description of laboratory analyses [40], Table S1: Linear regression analysis exploring the association between oxidative stress biomarkers regarding diet and smoking in the RBVD study, Table S2: Linear regression analysis exploring the association between oxidative stress biomarkers regarding diet and smoking in the Finland study, Table S3: Linear regression analysis exploring the association between oxidative stress biomarkers regarding diet and cyanoethyl mercapturic acid (CYMA) in the RBVD study, Table S4: Oxidative stress biomarkers according to vegan or omnivorous diet in the RBVD study using cyanoethyl mercapturic acid (CYMA) instead of smoking status as potential confounder, Table S5: Biomarkers of oxidative damage according to vegan or omnivorous diet of non-smoking participants of the RBVD and the Finnish study, Table S6: Linear regression analysis exploring the association between oxidative stress biomarkers regarding diet and sex in the RBVD study, Table S7: Linear regression analysis exploring the association between oxidative stress biomarkers regarding diet and sex in the Finland study, Table S8: Oxidative stress biomarkers according to vegan or omnivorous diet grouped by sex in the RBVD study, Figure S1: Scatterplot of oxidative damage biomarkers in the RBVD study, Figure S2: Scatterplot of oxidative damage biomarkers in the Finnish study, Figure S3: Scatter plots of the oxidative damage biomarkers and cyanoethyl mercapturic acid (CYMA) in the RBVD study.
