# Supplementary material for: Comparison of Five Oxidative Stress Biomarkers in Vegans and Omnivores from Germany and Finland
Source: Nutrients. 2022 Jul 16;14(14):2918. doi: 10.3390/nu14142918 (PMC9323774; doi:10.3390/nu14142918)
Supplement: Supplementary file 1 [file nutrients-14-02918-s001.zip › nutrients-1800112-supplementary.pdf]

Supplementary Materials

File S1: Detailed description of laboratory analyses

LC-Fluorescence analysis of MDA

MDA was detected by fluorescence after derivatization with thiobarbituric acid and separation by RP-HPLC as described by Wong et al. [17] with slight modifications [18]. In brief, 50 µl of plasma samples were incubated with thiobarbituric acid and phosphoric acid for 1 h at 95 °C. After neutralization with an equal volume of methanolic NaOH and centrifugation to remove possible residues, 20 µl were injected into a Shimadzu LC 20 System equipped with a Reprosil Pur 120 C18 AQ column (250 x 4.6 mm; 5 µm; Dr. Maisch GmbH). Excitation/emission of the fluorescence detector was set to 515 nm/553 nm. The run-time was 10 min per sample. Standards concentrations between 0.202 and 2.2 µmol/l were used and the standards were treated like samples.

Protein carbonyl ELISA

To prepare the standard curve we prepared reduced bovine serum albumin (BSA, containing very few carbonyl groups) and oxidized BSA (containing high concentrations of carbonyls). Reduced BSA was prepared by treating BSA with sodium borohydride followed by a dialysis step while oxidized BSA was prepared by treating BSA with hypochlorite. Carbonyl concentrations were measured spectrophotometrically at 375 nm after derivatization of reduced and oxidized BSA with 2,4-dinitrophenylhydrazine (DNPH), as described by Levine et al. [40]. Standards were stored until use at -80 °C. The protein concentrations of the samples were determined according to Bradford and the samples were then diluted to 4 mg protein/ml. Diluted plasma samples and standards were incubated with DNPH and then coated onto the wells of a 96-well plate (Nunc Immuno MaxiSorp) over night. The following day, the plate was blocked with reduced BSA in PBS. Following the primary antibody (anti-DNP, D9656 from Sigma) and the secondary antibody anti-rabbit (A1949 from Sigma) the plate was incubated with substrate solution containing o-phenylenediamine dihydrochloride (P1063 from Sigma) and the colour development stopped with H_2SO_4. The absorbance was measured at 492 nm (reference set to 750 nm) and the concentration was plotted against the standard concentrations [18].

3-Nitrotyrosine ELISA

To prepare the standard curve we prepared reduced BSA (containing very few nitrotyrosine groups) and nitrated BSA (containing high concentrations of nitro-groups). Reduced BSA was prepared by treating BSA with sodium hydrosuplhite, while nitrated BSA was prepared by treating BSA with peroxynitrite that was freshly prepared from mixing acidic hydrogen peroxide with sodium nitrite and sodium hydroxide. Nitrotyrosine concentrations were then measured spectrophotometrically at 438 nm. Standards were stored until use at -20 °C. The protein concentrations of the human plasma samples were determined according to a Bradford assay and the samples were diluted to 2 mg protein/ml. Diluted plasma samples and standards were coated onto the wells of a 96-well plate (Nunc Immuno MaxiSorp) over night. The following day, the plate was blocked with reduced BSA in PBS. Following the primary antibody (anti-Nitrotyrosine, 06-284 from Millipore) and the secondary antibody goat anti-rabbit (12-348 from Millipore) the plate was incubated with substrate solution containing o-phenylenediamine dihydrochloride (P1063 from Sigma) and the colour development stopped with H_2SO_4. The absorbance was measured at 492 nm (reference set to 750 nm) and the concentration was plotted against the standard concentrations [19].

LC-MS/MS Analysis of 8-hydroxy-2'-deoxyguanosine (8-OHdG)

After thawing the urine aliquots, 200 µl of each sample was centrifuged at 18.000xg for 5 min. Thirty µl of the supernatant were transferred to a glass vial with an 200 µl insert and diluted with 120 µl H_2O. Finally, 10 µl of an aqueous solution of $^{15}N_5$-8-OHdG (100 nM) were added and the sample was mixed thoroughly. 8-OHdG was analyzed with an Acquity I-Class UPLC (Waters) coupled to a QTrap 6500

mass analyzer (Sciex, Darmstadt, Germany) using a reversed-phase HSS T3 column (2 mm x 150 mm, 1.8 µm, Waters) and a gradient of ammonium formate (0.3 mM; solvent A) and acetonitrile (solvent B). The following gradient was applied: 0-3 min isocratic 97% A, 3-5 min linear from 97% to 94% A, 5-5.5 linear from 94% to 80% A, 5.5-5.6 min linear from 80% to 5% A, 5.6-7 min isocratic 5% A, 7-7.1 min linear from 5% to 97% A, 7.1-10 min isocratic 97% A. The flow rate was 300 µl/min and the injection volume was 5 µl. The column temperature was set to 40 °C. The mass spectrometer was operated in the positive ESI mode with the following parameters: curtain gas = 40 psi, ionspray voltage = 4500 V, entrance potential = 5 V, temperature = 450 °C, gas1 = 60 psi, gas2 = 70 psi. The 8-OHdG and $^{15}N_5$-8-OHdG were detected by multiple reaction monitoring (MRM) with the transitions *m/z* 284.1 to *m/z* 168.1 for the unlabeled and *m/z* 289.1 to *m/z* 173.1 for the $^{15}N_5$-labelled 8-OHdG with the following parameters: declustering potential = 50 V, collision energy = 23 V and cell exit potential = 8 V. The quantification of 8-OHdG was based on the signal intensity of $^{15}N_5$-8-OHdG.

LC-MS/MS Analysis of 8-isoprostaglandin F2α (8-iso-PGF2α)

8-Iso-PGF2α was extracted as described previously [20]. After thawing the urine, 2.7 ml of each sample was centrifuged at 2.500xg for 10 min. Aliquots of 2.5 ml were transferred to a new 15 ml reaction tube and 2 ml Tris-HCl (50 mM adjusted to pH 6 using 1N NH_3), 750 µl methanol and 5 µl of the standard d_4-8-iso-PGF2α (1 µM) were added. The analyte was enriched by solid-phase extraction (SPE) using Strata X-AW columns (100 mg, 3 ml, Phenomenex, Torrance, CA, USA) as follows. The SPE cartridges were equilibrated with 2 ml methanol containing 2% formic acid and 2 ml H_2O. After loading the samples, the cartridges were washed with 2 ml H_2O, 4 ml methanol/H_2O (1:4) and 2 ml acetonitrile. Thereafter, the cartridges were dried using a waterjet pump. The analyte was eluted with 2 x 1.5 ml methanol. The combined eluates were dried in a centrifugal evaporator at room temperature. The residues were dissolved in 50 µl methanol/H_2O (1:9), and centrifuged for 5 min at 18.000xg. The supernatant was transferred to a glass vial with a 200 µl insert. Analysis of 8-iso-PGF2α was done on an Acquity I-Class UPLC (Waters) coupled to a QTrap 6500 mass analyzer (Sciex). At this, the analytes were separated by reversed-phase chromatography on a HSS T3 column (2.0 × 150 mm, 1.8 µm, Waters) using a gradient of 0.3 mM ammonium formate (solvent A) and acetonitrile (solvent B). For the separation of 8-iso-PGF2α, the following gradient was used: 0-3 min isocratic 97% A, 3-5 min linear from 97% to 94% A, 5-9 min linear from 94% to 80% A, 9- 35 min linear from 80% to 55% A, 35-35.1 min linear from 55% to 5% A, 35.1-37 min isocratic 5% A, 37-37.1 min linear from 5% to 97% A, 37-40 min isocratic 97% A. The flow rate was 300 µl/min and the injection volume was 5 µl. The column temperature was set to 40 °C. The mass spectrometer was operated in the negative ESI mode with the following parameters: curtain gas = 40 psi, ionspray voltage = - 4500 V, entrance potential = - 10 V, temperature = 450 °C, gas1 = 60 psi, gas2 = 70 psi. The 8-iso-PGF2α and d_4-8-iso-PGF2α were detected by multiple reaction monitoring (MRM) with the transitions *m/z* 353.2 to *m/z* 193.2 for the unlabeled and *m/z* 357.2 to *m/z* 197.2 for the d_4-labeled 8-iso-PGF2α with the following parameters: declustering potential = - 200 V, collision energy = - 36 V and cell exit potential = - 11 V. The quantification of the intrinsic 8-iso-PGF2α was based on the signal intensity of d_4-8-iso-PGF2α.

LC-MS/MS analysis of CYMA

Urinary CYMA was analyzed after liquid-liquid extraction. The pH of 1 ml urine aliquots was adjusted by addition of 100 µl HCl (1 M). Ten µl d_3-CYMA (1 µM in MeOH/H_2O, 1:1) was added. The samples were mixed thoroughly and extracted with 1.5 ml ethyl acetate. After centrifugation (5 min, 3,500 rcf, room temperature), the extraction was repeated. The organic fractions were combined, 1 ml H_2O (pH 2, saturated with ethyl acetate) was added. After thorough mixing and centrifugation (5 min, 3,500 rcf, room temperature), the organic fraction was dried under reduced pressure. The residues were reconstituted in 100 µl of 50% MeOH. The samples were centrifuged (5 min, 18,000 rcf, room temperature) and the supernatant was transferred to glass vials.

CYMA was quantified using an HPLC 1100 (Agilent, Waldbronn, Germany) connected to a triple quadrupole-hybrid ion trap mass spectrometer QTrap6500 (Sciex, Darmstadt, Germany). The chromatographic separation was done by ion-pair reversed phase chromatography using a Nucleoshell RP 18plus (2.0 × 150 mm, 2.7 µm; Macherey–Nagel, Düren, Germany). The eluents were water

containing 10 mm tributylamine and 10 mm acetic acid (solvent A) and acetonitrile (solvent B). The flow rate of the gradient (0–1 min, 2% solvent B; 1–4 min, 2–36% solvent B; 4–8 min, 36–100% solvent B; 8–10 min, 100% solvent B; 10-10.1 min, 100-2% solvent B 10.1–13 min, 2% solvent B) was 0.5 mL min^{-1}. The temperature of the column oven was 40 °C and the sample injection volume was 5 µl. The operating parameters of the QTrap6500 were: ion spray voltage - 4500 V, interface heater temperature 450 °C, curtain gas 40 psi, ion source gas 1 60 psi, ion source gas 2 50 psi, collision activated dissociation gas set to medium. CYMA and d_3-CYMA were detected by multiple reaction monitoring (MRM) with the transitions *m/z* 215.0 to 162.0 and *m/z* 218.0 to 165.0 and the following parameters: declustering potential = - 30 V, entrance potential − 10 V, collision energy = - 12 V and cell exit potential = - 5 V. Data acquisition and processing were carried out using Analyst 1.7.1 software (Sciex, Darmstadt, Germany).

Supplemental tables and figures

Table S1: Linear regression analysis exploring the association between oxidative stress biomarkers regarding diet and smoking in the RBVD study.

Biomarker	Confounder	Estimate	Std. Error	t value	Pr(>|t|)
MDA [µmol/L]	Diet	0.03	0.11	0.30	0.77
	Smoking	0.33	0.16	2.02	**0.047**
	Diet*Smoking	-0.20	0.28	-0.73	0.47
	Model-Parameter	R^2= 0.02, F(3, 68)=1.51, p-value= 0.22			
Protein carbonyl [nmol/mg]	Diet	0.01	0.05	0.25	0.80
	Smoking	-0.06	0.08	-0.73	0.47
	Diet*Smoking	-0.06	0.13	-0.47	0.64
	Model-Parameter	R^2= -0.02, F(3, 68)=0.63, p-value=0.60			
3-Nitrotyrosine [pmol/mg]	Diet	-0.14	0.12	-1.16	0.25
	Smoking	-0.36	0.18	-1.99	0.051
	Diet*Smoking	0.17	0.30	0.54	0.59
	Model-Parameter	R^2=0.03, F(3, 68)=1.67, p-value=0.18			
8-iso-PGF2α [pmol/d]	Diet	-0.21	0.11	-2.01	0.05
	Smoking	-0.05	0.16	-0.35	0.73
	Diet*Smoking	0.34	0.26	1.28	0.20
	Model-Parameter	R^2=0.03, F(3, 68)=1.65, p-value=0.19			
8-OHdG [nmol/d]	Diet	-0.26	0.12	-2.11	0.04
	Smoking	-0.23	0.18	-1.25	0.21
	Diet*Smoking	0.47	0.31	1.51	0.13
	Model-Parameter	R^2= 0.03, F(3, 68)=1.67, p-value=0.18			

For analysis oxidative stress biomarkers were log-transformed to fit normality assumption.

Table S2: Linear regression analysis exploring the association between oxidative stress biomarkers regarding diet and smoking in the Finland study.

Biomarker	Confounder	Estimate	Std. Error	t value	Pr(>|t|)
MDA [µmol/L]	Diet	-0.24	0.12	-2.03	0.05
	Smoking	-0.17	0.21	-0.84	0.40
	Diet*Smoking	-0.15	0.32	-0.46	0.65
	Model-Parameter	R^2= 0.11, F(3, 33)=2.42, p-value= 0.08			
Protein carbonyl [nmol/mg]	Diet	-0.03	0.06	-0.52	0.60
	Smoking	0.12	0.10	1.20	0.24
	Diet*Smoking	-0.08	0.16	-0.53	0.60
	Model-Parameter	R^2= -0.02, F(3, 33)=0.81, p-value=0.50			
3-Nitrotyrosine [pmol/mg]	Diet	-0.50	0.17	-2.90	**0.007**
	Smoking	-0.05	0.31	-0.15	0.89
	Diet*Smoking	-0.22	0.47	-0.46	0.65
	Model-Parameter	R^2=0.18, F(3, 33)=3.69, p-value=**0.02**			

Table S3: Linear regression analysis exploring the association between oxidative stress biomarkers regarding diet and cyanoethyl mercapturic acid (CYMA) in the RBVD study.

Biomarker	Confounder	Estimate	Std. Error	t value	Pr(>|t|)
MDA [µmol/L]	Diet	0.05	0.0999	0.4657	0.64
	CYMA	0.0033	0.0009	3.7747	**0.0003** [a]
	Diet*CYMA	-0.0021	0.0016	-1.2925	0.20
	Model-Parameter	R^2=0.15, F(3, 68)=5.09, p-value= 0.003			
Protein carbonyl [nmol/mg]	Diet	0.03	0.05	0.50	0.62
	CYMA	-0.0001	0.0004	-0.32	0.75
	Diet*CYMA	-0.001	0.001	-0.84	0.40
	Model-Parameter	R^2=-0.02, F(3, 68)=0.56, p-value=0.65			
3-Nitrotyrosine [pmol/mg]	Diet	-0.09	0.12	-0.77	0.44
	CYMA	-0.001	0.001	-1.27	0.21
	Diet*CYMA	0.0001	0.002	0.05	0.96
	Model-Parameter	R^2=-0.01, F(3, 68)=0.89, p-value=0.45			
8-iso-PGF2α [pmol/d]	Diet	-0.19	0.10	-1.81	0.07
	CYMA	0.0004	0.001	0.45	0.65
	Diet*CYMA	0.001	0.002	0.79	0.43
	Model-Parameter	R^2=0.03, F(3, 68)=1.61, p-value=0.20			
8-OHdG [nmol/d]	Diet	-0.26	0.12	-2.23	0.03
	CYMA	-0.002	0.001	-1.84	0.07
	Diet*CYMA	0.003	0.002	1.76	0.08
	Model-Parameter	R^2=0.05, F(3, 68)=2.27, p-value=0.09			

[a]After exclusion of one omnivore with MDA concentrations higher than 6 µmol/L, the Pr(>|t|) of CEMA was 0.31.

Table S4: Oxidative stress biomarkers according to vegan or omnivorous diet in the RBVD study using cyanoethyl mercapturic acid (CYMA) instead of smoking status as potential confounder.

Biomarker	Vegans (n=36)	Omnivores (n=36)	*p-value*
MDA [µmol/L]	1.08 (0.95-1.24)	1.04 (0.91-1.19)	**0.03**[a]
Protein carbonyls [nmol/mg]	0.81 (0.76-0.87)	0.81 (0.76-0.87)	0.25
3-nitrotyrosine [pmol/mg]	2.43 (2.08-2.84)	2.83 (2.42-3.31)	0.21
8-OHdG [nmol/d]	13.3 (11.4-15.5)	16.1 (13.8-18.7)	0.052
8-iso-PGF2α [pmol/d]	666 (588-754)	738 (652-835)	**0.003**

Oxidative stress biomarkers are reported as geometric mean (95%-CI). The model was adjusted for age, sex, BMI, CYMA, PA, alcohol consumption, total energy intake. [a]After exclusion of one omnivore with MDA concentrations higher than 6 µmol/L the difference in MDA concentrations between vegans and omnivores was no longer significant.

Table S5. Biomarkers of oxidative damage according to vegan or omnivorous diet of non-smoking participants of the RBVD and the Finnish study.

Biomarker	Model	RBVD study Vegans (n=32)	RBVD study Omnivores (n=27)	RBVD study p-value	Finnish study Vegans (n=19)	Finnish study Omnivores (n=13)	Finnish study p-value
MDA [µmol/L]	1	1.03 (0.91-1.17)	1.00 (0.87-1.14)	0.73	0.82 (0.70-0.96)	1.03 (0.86-1.25)	0.06
	2	1.03 (0.90-1.17)	0.99 (0.86-1.14)	0.95	0.82 (0.70-0.96)	1.03 (0.85-1.24)	0.14
	3 [a]	1.02 (0.90-1.17)	1.00 (0.87-1.15)	0.93	0.81 (0.70-0.94)	0.99 (0.81-1.22)	0.25
Protein carbonyl [b] [nmol/mg]	1	0.83 (0.77-0.89)	0.82 (0.76-0.89)	0.81	0.95 (0.88-1.02)	0.99 (0.90-1.07)	0.52
	2	0.83 (0.77-0.89)	0.82 (0.75-0.89)	0.90	0.95 (0.88-1.02)	0.99 (0.90-1.08)	0.71
	3 [a]	0.82 (0.76-0.88)	0.83 (0.77-0.90)	0.15	0.95 (0.88-1.01)	0.93 (0.83-1.02)	0.64
3-nitrotyrosine [pmol/mg]	1	2.58 (2.20-3.03)	2.97 (2.50-3.54)	0.24	1.86 (1.49-2.31)	3.07 (2.36-4.00)	**<0.01**
	2	2.55 (2.17-2.99)	3.02 (2.53-3.59)	0.37	1.86 (1.49-2.33)	3.06 (2.33-4.01)	**0.04**
	3 [a]	2.54 (2.15-3.00)	3.03 (2.53-3.64)	0.67	1.87 (1.55-2.26)	3.12 (2.40-4.06)	**<0.01**
8-OHdG [nmol/d]	1	13.0 (11.1-15.3)	16.9 (14.2-20.1)	**0.03**	-	-	-
	2	13.2 (11.3-15.5)	16.6 (14.0-19.8)	**0.03**	-	-	-
	3	13.2 (11.3-15.6)	16.6 (13.9-19.8)	0.10	-	-	-
8-iso-PGF2α [pmol/d]	1	625 (541-722)	772 (660-904)	0.05	-	-	-
	2	639 (562-726)	752 (654-864)	**<0.001**	-	-	-
	3	645 (568-733)	744 (646-855)	**<0.01**	-	-	-

Oxidative stress biomarkers are reported as geometric mean (95%-CI). [a] In the Finnish study five omnivores had missing values of confounders and were excluded in Model 3. [b] In the Finnish study, protein carbonyl values represent arithmetic means. Model 1: unadjusted; Model 2: adjusted for age, sex, BMI; Model 3: adjusted for age, sex, BMI, smoking status, physical activity, alcohol consumption, total energy intake; Abbreviation: MDA, malondialdehyde; 8-OHdG, 8-hydroxy-2'-deoxyguanosine; 8-iso-PGF2α, 8-iso-prostaglandin F2α.

Table S6: Linear regression analysis exploring the association between oxidative stress biomarkers regarding diet and sex in the RBVD study.

Biomarker	Confounder	Estimate	Std. Error	t value	Pr(>|t|)
MDA [µmol/L]	Diet	0.13	0.14	0.90	0.37
	Sex	0.20	0.14	1.44	0.16
	Diet*Sex	-0.33	0.20	-1.63	0.11
	Model-Parameter	R^2= -0.001, F(3, 68)=0.98, p-value= 0.41			
Protein carbonyl [nmol/mg]	Diet	0.08	0.07	1.16	0.25
	Sex	0.00	0.07	-0.05	0.96
	Diet*Sex	-0.12	0.09	-1.33	0.19
	Model-Parameter	R^2= 0.01, F(3, 68)=1.29, p-value=0.29			
3-Nitrotyrosine [pmol/mg]	Diet	-0.07	0.16	-0.42	0.67
	Sex	-0.04	0.16	-0.24	0.81
	Diet*Sex	-0.01	0.23	-0.05	0.96
	Model-Parameter	R^2=-0.04, F(3, 68)=0.19, p-value=0.90			
8-iso-PGF2α [pmol/d]	Diet	-0.17	0.12	-1.38	0.17
	Sex	0.35	0.12	2.86	**0.01**
	Diet*Sex	0.00	0.17	0.03	0.98
	Model-Parameter	R^2=0.20, F(3, 68)=6.77, p-value=0.0005			
8-OHdG [nmol/d]	Diet	-0.28	0.15	-1.83	0.07
	Sex	0.19	0.15	1.24	0.22
	Diet*Sex	0.20	0.21	0.93	0.35
	Model-Parameter	R^2= 0.10, F(3, 68)=3.60, p-value=0.02			

Table S7: Linear regression analysis exploring the association between oxidative stress biomarkers regarding diet and sex in the Finland study.

Biomarker	Confounder	Estimate	Std. Error	t value	Pr(>|t|)
MDA [µmol/L]	Diet	-0.39	0.13	-3.08	**0.004**
	Sex	-0.31	0.15	-2.15	**0.038**
	Diet*Sex	0.46	0.21	2.21	**0.034**
	Model-Parameter	R^2= 0.15, F(3, 35)=3.31, p-value= 0.03			
Protein carbonyl [nmol/mg]	Diet	-0.06	0.07	-0.94	0.35
	Sex	-0.01	0.08	-0.19	0.85
	Diet*Sex	0.04	0.11	0.36	0.72
	Model-Parameter	R^2= -0.10, F(3, 35)=0.34, p-value=0.80			
3-Nitrotyrosine [pmol/mg]	Diet	-0.46	0.19	-2.37	0.024
	Sex	0.05	0.23	0.21	0.83
	Diet*Sex	-0.11	0.32	-0.34	0.74
	Model-Parameter	R^2=0.17, F(3, 35)=3.59, p-value=**0.02**			

Table S8: Oxidative stress biomarkers according to vegan or omnivorous diet grouped by sex in the RBVD study.

Biomarker	Sex	Vegans	Omnivores	p-value
MDA [µmol/L]	women	1.11 (0.94-1.31)	0.98 (0.82-1.15)	0.28
	men	0.98 (0.78-1.24)	1.2 (0.95-1.51)	0.23
Protein carbonyl [nmol/mg]	women	0.87 (0.80-0.95)	0.81 (0.74-0.88)	0.23
	men	0.77 (0.69-0.85)	0.81 (0.73-0.89)	0.50
3-Nitrotyrosine [pmol/mg]	women	2.59 (2.02-3.32)	2.77 (2.16-3.56)	0.70
	men	2.46 (2.00-3.03)	2.67 (2.17-3.28)	0.59
8-iso-PGF2α [pmol/d]	women	540 (457-638)	640 (542-756)	0.15
	men	770 (640-926)	907 (755-1091)	0.21
8-OHdG [nmol/d]	women	11.1 (8.8-13.9)	14.6 (11.6-18.3)	0.10
	men	16.2 (13.3-19.9)	17.5 (14.3-21.5)	0.59

Expressed as geometric mean (95%-CI), unadjusted; each diet and sex group contained 18 participants

FIGURES below

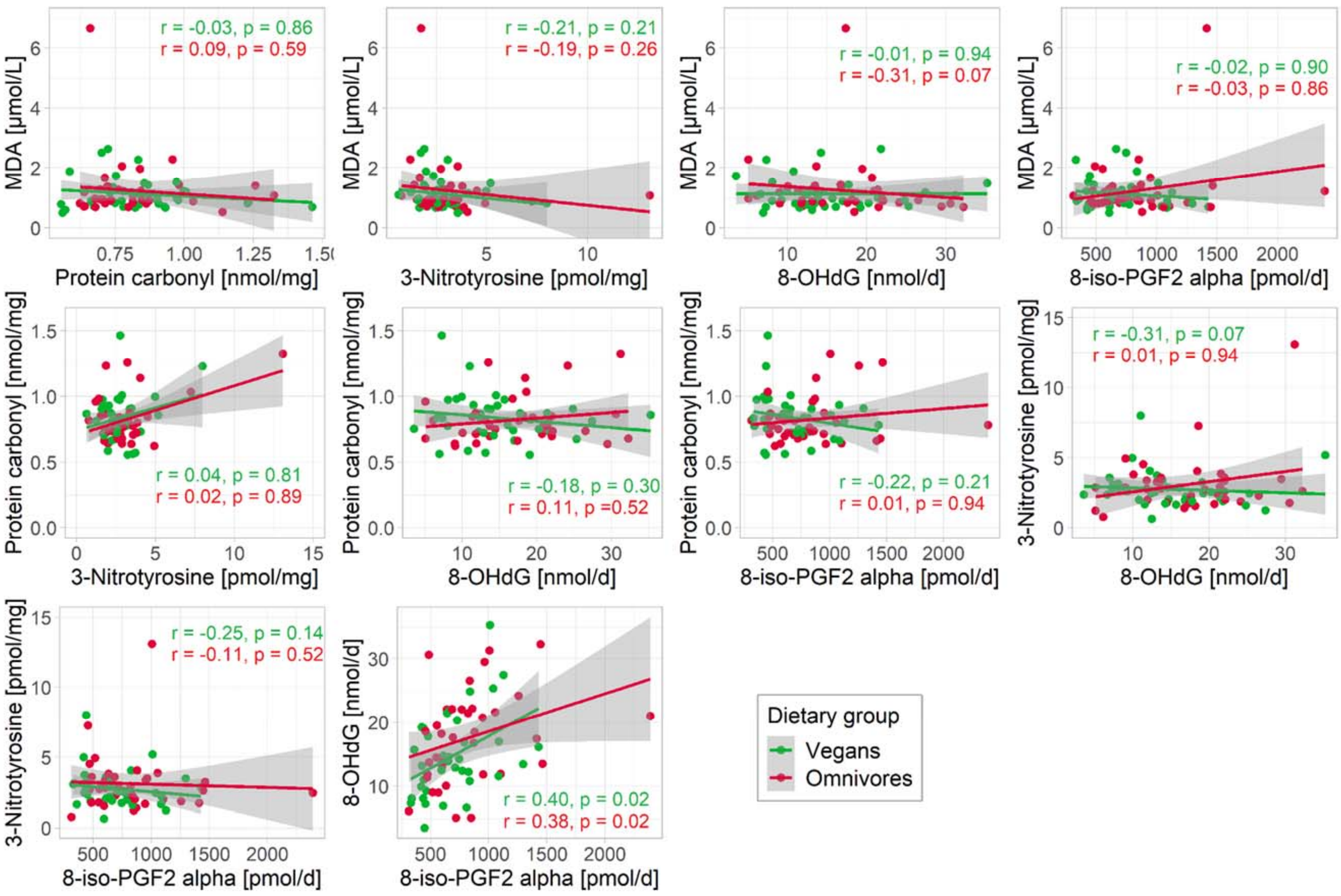

Figure S1: Scatterplot of oxidative damage biomarkers in the RBVD study.

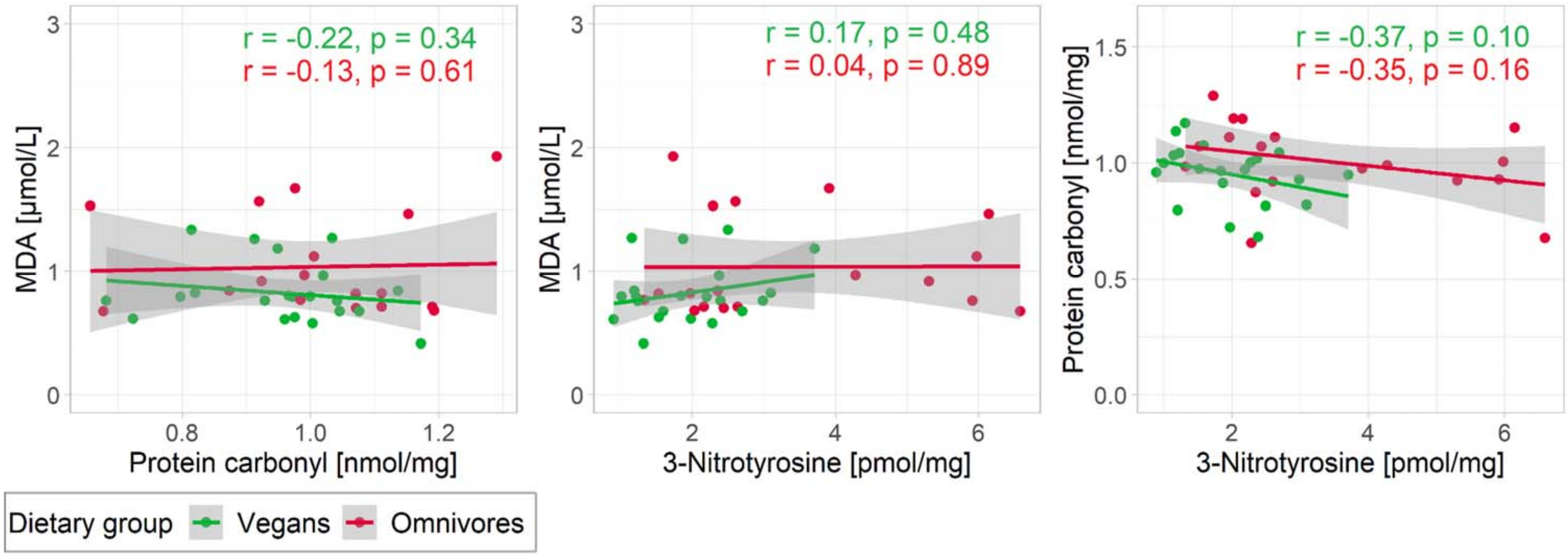

Figure S2: Scatterplot of oxidative damage biomarkers in the Finish study.

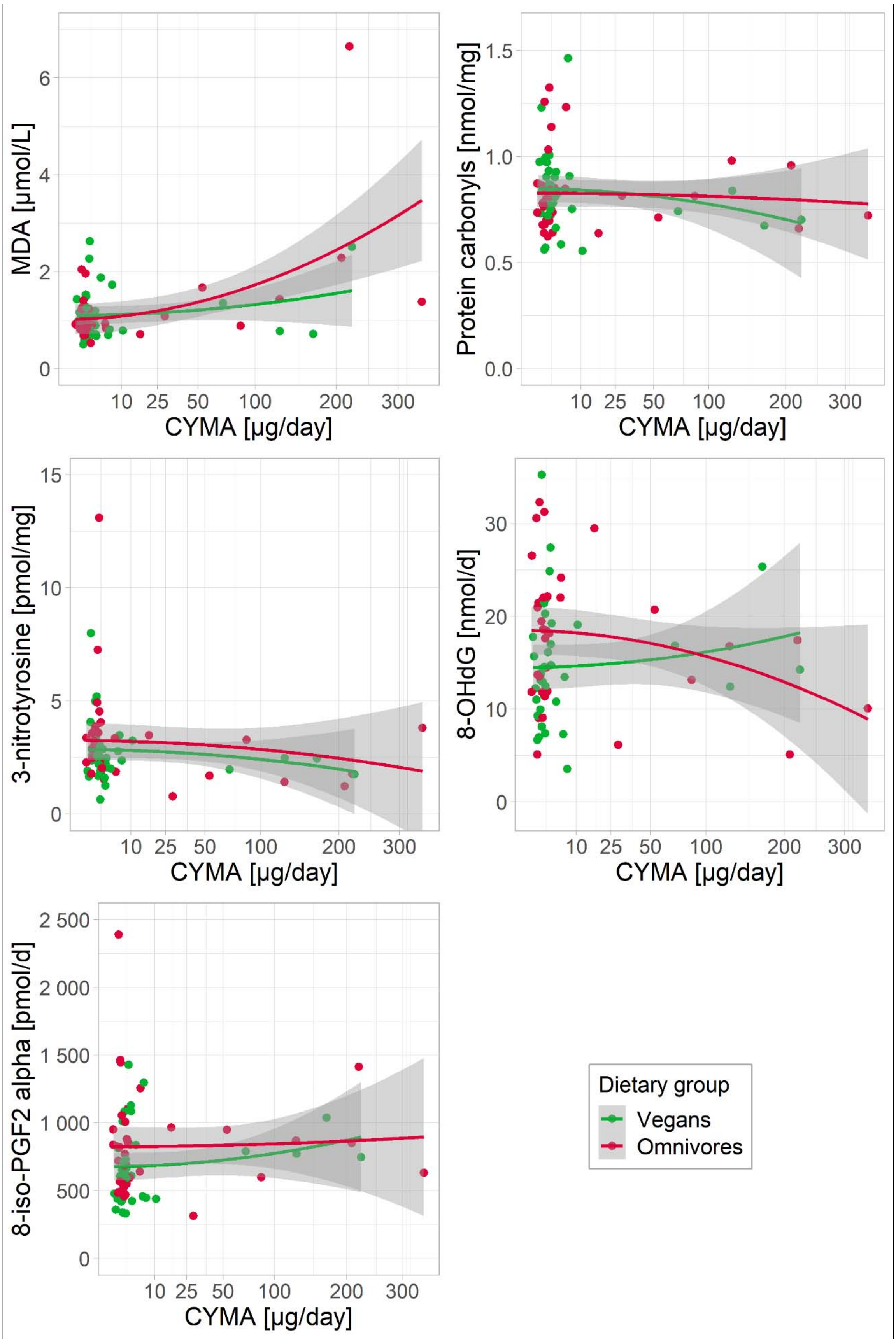

Figure S3: Scatter plots of the oxidative damage biomarkers and cyanoethyl mercapturic acid (CYMA) in the RBVD study.
